# Mapping the nanoscale elastic property modulations of polypyrrole thin films in liquid electrolyte with EC-AFM†

**DOI:** 10.1039/d3na00611e

**Published:** 2023-11-22

**Authors:** Alexander Meinhardt, Pirmin Lakner, Patrick Huber, Thomas F. Keller

**Affiliations:** a Centre for X-ray and Nano Science (CXNS), Deutsches Elektronen-Synchrotron DESY Hamburg Germany alexander.meinhardt@desy.de thomas.keller@desy.de; b Department of Physics, Hamburg University Hamburg Germany; c Hamburg University of Technology, Institute for Materials and X-Ray Physics Hamburg Germany; d Center for Hybrid Nanostructures CHyN, Hamburg University Hamburg Germany

## Abstract

Linking structure to mechanical and elastic properties is a major concern for the development of novel electroactive materials. This work reports on the potential-induced changes in thickness and Young modulus of a substrate supported, perchlorate doped polypyrrole thin film (<100 nm) investigated with electrochemical atomic force microscopy (AFM) under *in situ* conditions. This was accomplished by nanomechanical mapping of potentiodynamically electropolymerized polypyrrole film in electrolyte solution with AFM during redox cycling. The polypyrrole film thickness and Young modulus follow the electrical potential nearly linearly, increasing due to solvent and ion influx as the film is oxidized, and decreasing during reduction. Our measurements also confirm the presence of a potential-independent, passive swelling which is accompanied by softening of the film, likely caused by osmotic effects. Additionally, the heterogeneous distribution of the Young modulus can be directly traced to the typical nodular surface topography of polypyrrole, with the top of the nodular area possessing lower modulus, thus highlighting the complex relationship between topography and elastic properties.

## Abstract 2

Linking structure to mechanical and elastic properties is a major concern for the development of novel electroactive materials.

## Graphical abstract

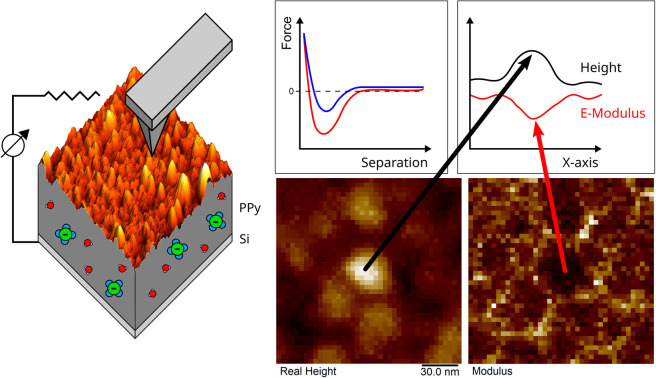

## Introduction

Conductive polymers such as polypyrrole (PPy) have received a lot of interest due to their potential in a wide range of applications. Their high electrical conductivity, stability, and mechanical response when subjected to applied electrical potentials make them ideal candidates for applications in microactuators, sensors, supercapacitors, and many more.^1–9^ The volume expansion/contraction PPy undergoes during reversible oxidation and reduction is associated with the ion (and/or solvent) diffusion into and out of the polymer matrix.^8,10–12^ It is well known that perchlorate doped PPy shows largely anion-dominant ion transport properties and experiences anion influx to the polymer matrix upon oxidation, leading to volume expansion.^2^ Since the kinetics of these doping–undoping processes and the electrochemical responses are highly dependent on the electrochemical surface properties, characterizing nanoscale variations in the interfacial properties such as topography, adhesion, and elasticity is an important factor when considering possible influence on interactions with other interfaces when designing applications.^3,12–15^ PPy offers the advantage of combining low operating voltages with good biocompatibility while showing electrochemical actuation comparable to, or larger than piezo ceramics, making it an attractive candidate for biomedical actuators. Recently, a study performed on a nanoporous silicon-PPy composite membrane concluded that although the macroscopic behavior of these actuation properties is well understood, there still remain fundamental questions on the influence of the systems' microstructure on its macroscopic properties.^7,8^ However, this issue is not exclusive to hybrid systems, but also applies to other sample geometries, *e.g.* thin films.

AFM has emerged as a versatile and powerful tool to not only characterize the surface morphology and topography, but also to simultaneously measure and map the elastic properties of polymer surfaces.^14–23^ A cantilever with a sharp tip can reliably sense tip–sample interaction forces in the piconewton regime, enabling the investigation of polymers and soft biological samples under liquid environment conditions.^24,25^ Force–distance curves are commonly recorded as the result of probing the sample surface with a cantilever tip by nanoindentation. Analysis of those curves offers an insight into the elastic properties of polymers and soft matter. Utilizing the tip–surface interactions to record a nanometer-scale 2D mapping of the mechanical and topographical properties of the surface provides unique means of linking structure to properties, thus opening pathways for the development of more advanced materials.^17^

It is well known that the elastic properties of PPy can be influenced by many factors, such as the type of dopant salt, solvent, synthesis, and many more. Utilizing these factors, Young's moduli ranging from 0.03 GPa up to 1.50 GPa have been achieved.^26^ For a perchlorate-doped, freestanding PPy film, a Young's modulus of up to 500 MPa has been recorded by macroscopic tensile strength tests.^27^ The film showed a linear relationship between charge, strain, and elastic modulus during redox cycling. As the PPy film was oxidized at higher potentials, it experienced hardening due to solvent and perchlorate induced swelling. While macroscopic tensile tests offer useful insight about the global behavior of the PPy film, they lack information about the influence of the PPy film surface properties on its electrochemical behavior. Mapping the mechanical and topographical properties of PPy thin films *via* nanoindentation with AFM under electrochemical conditions is necessary to unravel the complex relationship between surface structure and properties of conductive PPy thin films.

Therefore, this study aims to illuminate the complex relationship between topography and mechanical properties of perchlorate doped thin PPy films (<100 nm) during redox cycling with *in situ* EC-AFM. Hereby we focus on the potential induced changes in film thickness and elastic modulus determined from fast-recorded nanoindentation curves. We also observe a potential independent change likely caused by osmotic effects.

## Experimental

### Materials and methods

All chemicals were purchased from Sigma-Aldrich. Pyrrole was distilled prior to use. Ultrapure water was taken from a water purification system (18.2 MΩ m). The potentiodynamic electropolymerization and subsequent *in situ* experiments were performed using a potentiostat with a three-electrode setup. All potentials are shown relative to the reference electrode.

### Substrate preparation

The sample substrate consisted of a p-doped (100)-oriented silicon wafer. To remove the native silicon oxide layer on the silicon wafer, the wafer was first immersed in an aqueous solution of 40% ammonium fluoride and 0.05 M ammonium sulfite for five minutes and then rinsed with water and dried under nitrogen flow.

### Polypyrrole electropolymerization

The substrate was fixed in a custom electrochemical cell for electropolymerization of PPy (Fig. S1†). The cell utilized a three-electrode setup containing the substrate as working electrode (WE), a Pt wire as counter electrode (CE), and an Ag/AgCl electrode as reference electrode (RE). The cell was then filled with acetonitrile solution of 0.1 M pyrrole and 0.1 M lithium perchlorate. Potentiodynamic electropolymerization was performed by cycling the potential 200 times between 0.3 V and 1.1 V at a potential scanning rate of 1 V s^−1^. After electropolymerization, the PPy film was carefully rinsed with water and transferred to the AFM.

### AFM (in air, in liquid/EC-AFM, force spectroscopy)

The AFM measurements were performed using a Dimension Icon AFM (Bruker).^28^ Small areas of the PPy film were scratched off with tweezers to act as a planar reference during the experiments (Fig. S3 and S7†). Measurements in air were performed using standard tapping cantilevers (RTESPA-300, Bruker). 1 × 1 μm scans were recorded in tapping mode near the substrate with a resolution of 256 × 256 pixels and a scan rate of 0.5 Hz.

For the potential dependent *in situ* experiments, the PPy film was placed in a fluid cell and connected to a three-electrode setup, with the substrate as working electrode and Cu wires fixed above the sample as counter electrode and reference electrode (Fig. S2†). The film was then covered with 0.1 M sodium perchlorate solution (aq.) and given a few minutes to stabilize before the start of the measurements. Silicon nitride cantilevers were employed during the *in situ* measurements (SCANASYST-FLUID+, Bruker) and a splash shield cover was used to minimize electrolyte evaporation. The measurements were performed using the ‘Electrochemistry PeakForce Quantitative NanoMechanics’ (EC-QNM) mode of the Dimension Icon. PeakForce EC-QNM enables the measurement of elastic properties while applying electrical potentials and performing fast force–distance curves. The sample was first imaged with no potential applied by scanning a 1 × 1 μm area near the substrate with a resolution of 512 × 512 pixels at a scan rate of 0.5 Hz.

The PPy film was then investigated at different electrical potentials. Constant potentials ranging from 0.0 V to 0.3 V were applied and the sample was imaged after pausing a few minutes after each potential change to account for non-equilibrium effects. A series of 5 × 5 μm scans with a resolution of 256 × 256 pixels and a scan rate of 0.5 Hz were taken as shown in Fig. S5.† This technique will be referred to as ‘static’ method below.

Additionally, cyclic voltammetry was performed while imaging the PPy film. A periodic triangular potential between 0.1 V and 0.3 V with a potential scanning rate of 10 mV s^−1^ was applied. During electrochemical cycling the AFM slow scan axis was disabled, which resulted in the AFM tip repeatedly scanning the same line, thus enabling direct tracking of the potential dependent film thickness and elastic modulus evolution. The principle is shown in Fig. S6.† Line profiles of 1 μm length with a resolution of 512 pixels were continuously collected at a scan rate of 1 Hz over the duration of several redox cycles. This method will be referred to as ‘dynamic’ method below. The film remained stable for several hours within the electrolyte. After excessive potential cycling, it showed signs of partial delamination.

The PPy film was then stored in air for about a year. Subsequently, the sample's elastic properties were investigated with the QNM mode of the Dimension Icon. A standard tapping cantilever was used (RTESPA-300, Bruker) and calibrated beforehand, yielding a spring constant of 22.7 N m^−1^ and a deflection sensitivity of 83.3 nm V^−1^. A force setpoint of 100 nN was used during the simultaneous mapping of PPy film topography, reduced elastic modulus, and adhesion. A systematically optimized force setpoint was chosen to ensure a sufficient indentation depth while maintaining film integrity as seen in Fig. S8.† For a force setpoint >100 nN, the image contrast got worse, indicating sample or tip damage. The tip radius was determined to be 34 nm after optimization of the force setpoint and was used as an upper limit for the tip radius, while the actual resolution at 100 nN was much better.

## Results

### Electropolymerization of polypyrrole

**Fig. 1:**
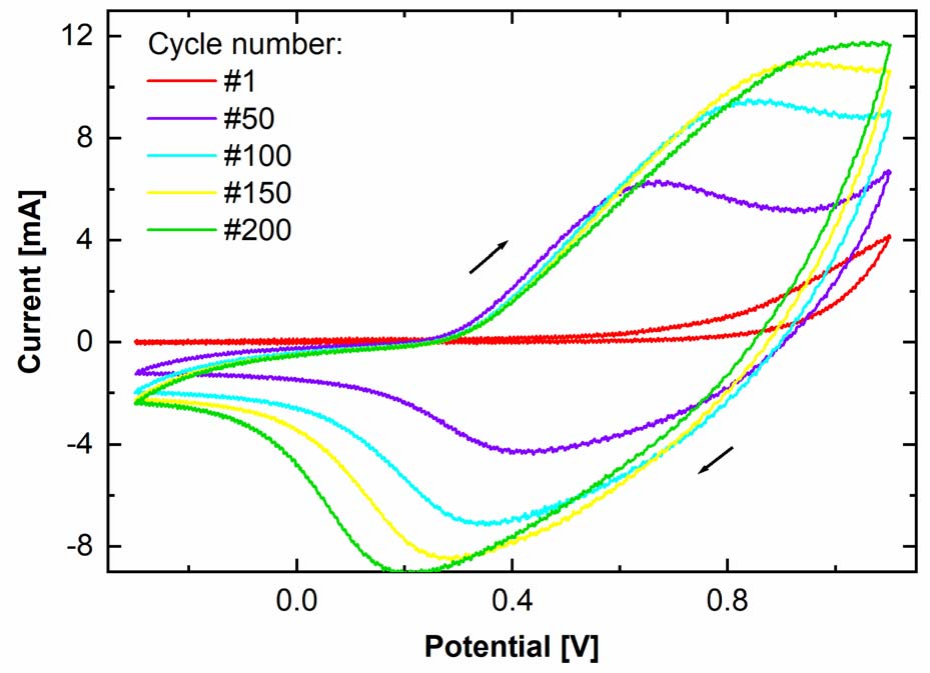
Cyclic voltammogram of the PPy electrodeposition for 200 cycles. Shown are the first, 50th, 100th, 150th, and 200th cycle.

A thin film of perchlorate doped PPy was deposited *via* potentiodynamic electropolymerization. The used deposition protocol is known to produce dense, adherent films with low porosity and high conductivity.^4,6,29,30^ In Fig. 1, the cyclic voltammogram of the PPy electropolymerization shows how the current, and correspondingly the transferred charge originating from oxidization and reduction of the growing film increase each cycle. The increase of the area enclosed by the cyclic voltammogram per cycle indicates continuous film growth. The transferred charge during synthesis is determined to be 171 ± 3 mC by integration of the area enclosed by the cyclic voltammogram. By utilizing standard literature growth rates,^9,31,32^ a PPy film thickness of 44.5 ± 2.7 nm is calculated.

### AFM in air & electrolyte

**Fig. 2:**
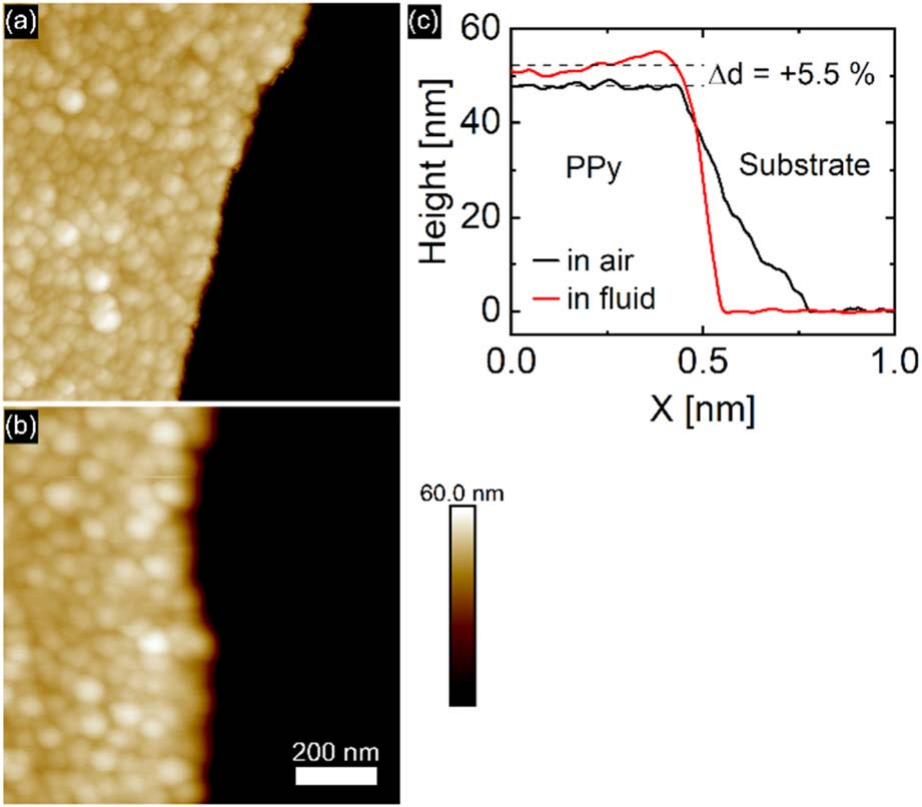
PPy topography in air (a) and when immersed in perchlorate solution (b) recorded with AFM. (c) Comparison of average PPy height profiles between air and fluid environment, showing a solvent induced swelling of 5.5%.

After synthesis, the PPy film was studied in air with AFM tapping mode, revealing the typical cauliflower PPy surface topography made up of globular areas of varying sizes. The film thickness was measured by scanning along the free-scraped substrate area and the PPy film as shown in Fig. 2. Using the free scratched substrate area as baseline, an average PPy film thickness of 48.4 ± 2.1 nm is determined, a value in good agreement with the film thickness calculated from the transferred charge during electropolymerization.

Solvent induced height changes up to 70% have been reported in the literature.^2,11,26^ Recent reports of PPy electropolymerized under similar conditions as ours reported a swelling ratio of 3.7% upon submersion of the film in perchloric acid.^6^ In our case, immersion of the PPy film in 0.1 M (aq.) NaClO_4_ solution was accompanied by an 5.5% increase in film thickness compared to the dry state caused by solvent intake visible in Fig. 2c. The absence of protons in neutral aqueous electrolyte solutions usually leads to a higher swelling capacity, since no protons can partake in charge compensation.^6^ While there are many factors that influence the rate of PPy swelling in electrolyte environment, it is generally known that PPy electropolymerized in non-aqueous media and deposited under potential control produces dense and highly conductive PPy films.^6,29,30^ Additionally, the use of large, bulky anions (*e.g.* perchlorate, sulfonate) yields PPy films with low roughness and high homogeneity.^33^ The PPy film roughness changed from 2.3 nm to 2.8 nm upon immersion of the film in electrolyte.

### EC-QNM AFM (static/dynamic method)

As polymer relaxation, electric transport, and ion and solvent diffusion all take place on different time scales during electrochemical potential cycling of PPy, it is necessary to use a static method, where the film is assumed to be in equilibrium while applying a constant electrical potential during the AFM measurements, as well as a dynamic method, where potential is varied while changes of the PPy topography and elastic properties are recorded as a function of electric potential. This gives access to the different processes involved during the potential cycling of the PPy sample.

**Fig. 3:**
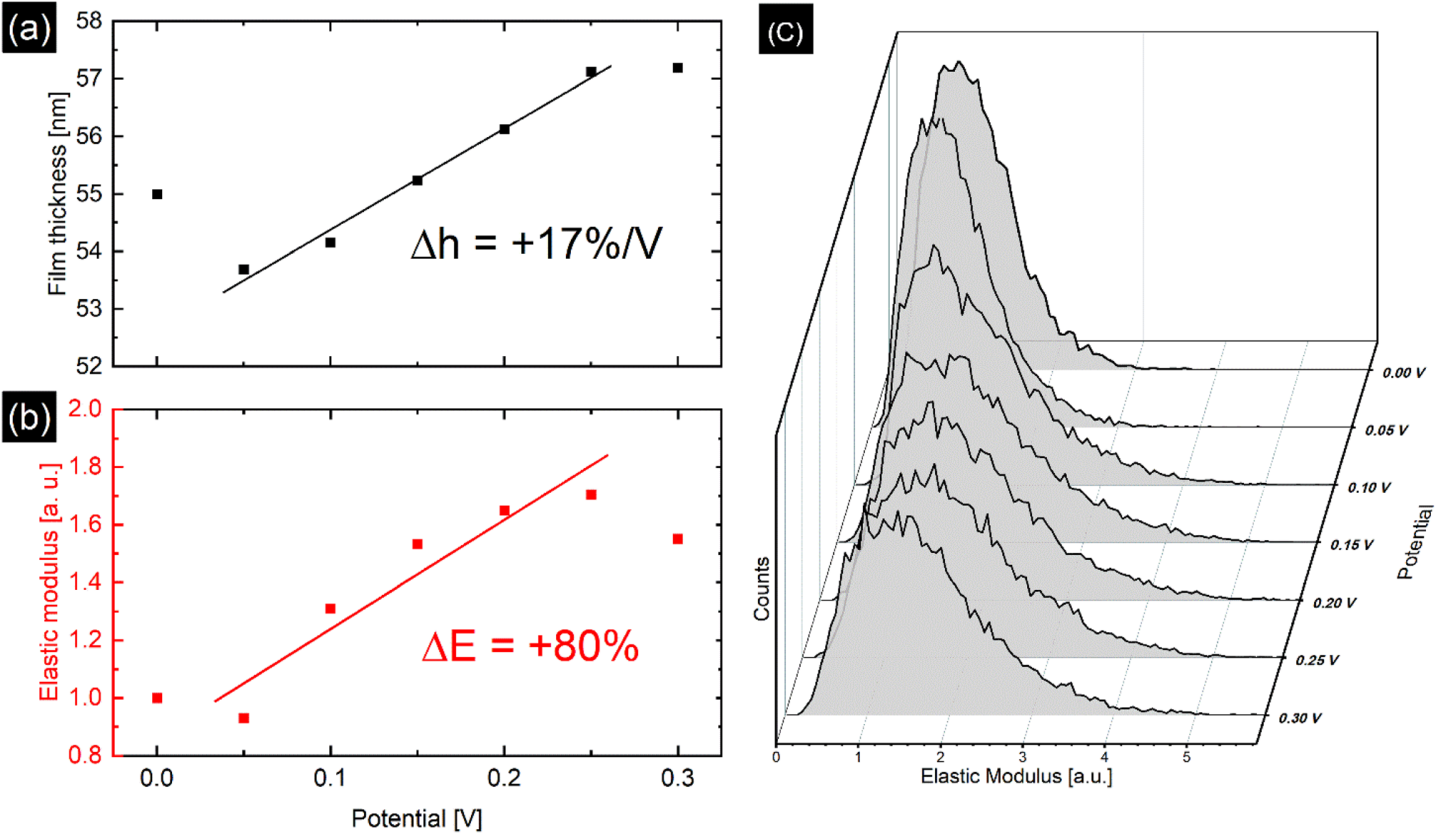
(a) Potential dependent film thickness measured *via* static method with linear fit, yielding a swelling ratio of 17% V^−1^. (b) Relative changes in Young modulus normalized with respect to the modulus at 0.0 V with linear fit, showing an increase of 80%. The deviation from the linear trend seen at 0 V and 0.3 V is attributed to nonequilibrium effects such as chain relaxation. (c) Distribution of elastic modulus of the PPy area marked in Fig. S5† for every applied potential. The modulus distribution shifts and widens under oxidizing conditions.

With the static method, the PPy film was investigated in perchlorate electrolyte with EC-QNM AFM at defined potential steps ranging from 0.0 V to 0.3 V with enough time (1–2 minutes) given for the film to adjust to the applied potential before imaging. Image drift shows the same tendency at every potential. All topographic data is accordingly drift-corrected by 1st order plane fitting in *x*- and *y*-axis on the exposed substrate area. Then the average height and elastic modulus of the film are determined at every potential step as shown in Fig. 3. As the PPy is oxidized, the film expands, yielding a swelling ratio of 17% V^−1^.

**Fig. 4:**
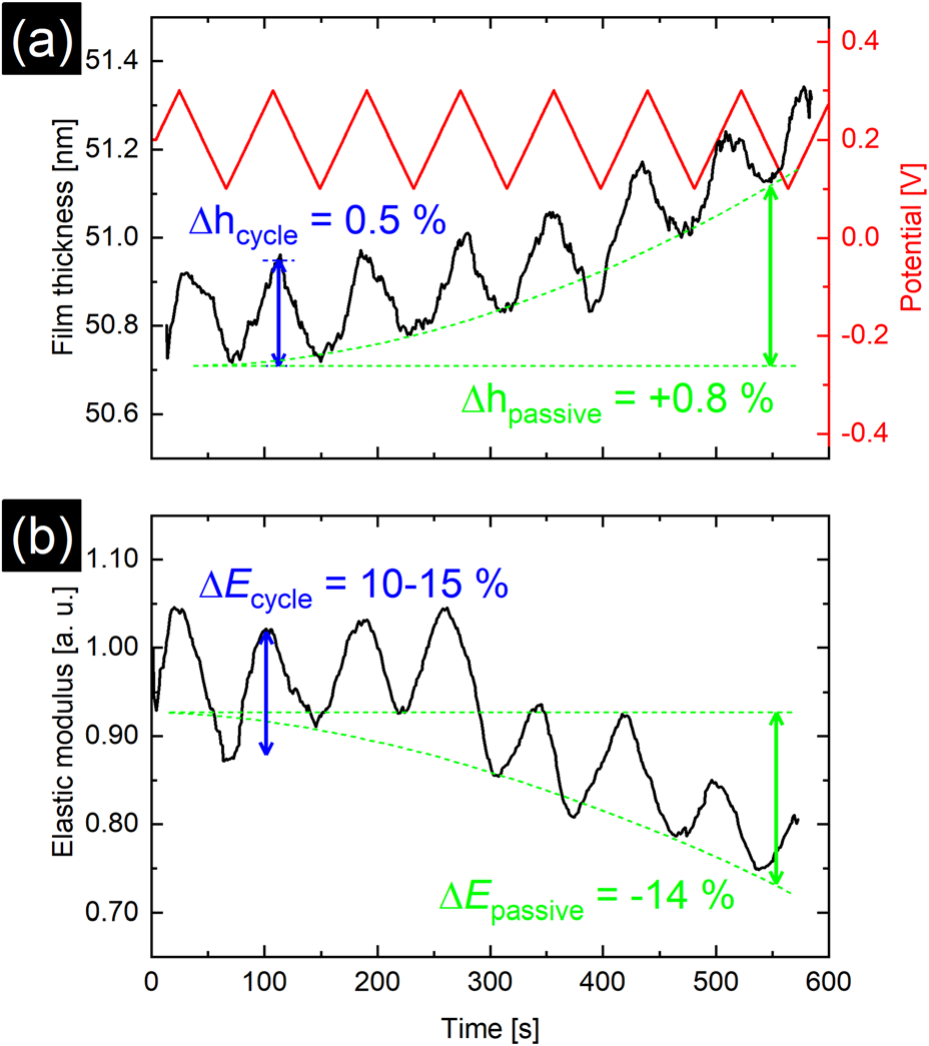
(a) Dynamic method results for film thickness over time. Periodic triangular potential ranging from 0.1 V to 0.3 V with a potential cycling rate of 10 mV s^−1^ over time is shown in red. (b) Evolution of average reduced elastic modulus over time. Both film thickness and elastic modulus closely follow the applied potential. Additionally, a potential independent swelling, coupled with softening of the PPy film over several redox cycles is visible.

In order to monitor the topographical and elastic changes of the PPy film at shorter timescales, a setup similar to earlier investigations of PPy is used.^2,11,13^ Fast processes taking place at shorter timescales include ion and solvent diffusion into and out of the PPy film for charge compensation upon oxidation and reduction and changes in PPy backbone such as bond length, chain conformation, and interchain interactions, whereas effects such as osmotic expansion caused by osmotic pressure are more pronounced at longer timescales.^6,11,34,35^ The ‘dynamic’ method utilizes cyclic voltammetry and AFM simultaneously. The issue of time-limitation due to the scanning probe working principle of AFM is eliminated by disabling the slow scan axis as illustrated in Fig. S6.† A continuous series of line profiles was recorded while cycling the electrical potential from 0.1 V to 0.3 V with a cycling rate of 10 mV s^−1^. The results shown in Fig. 4a reveal potential induced thickness changes of 0.5–0.6% per cycle (2.5–3.0% V^−1^ swelling ratio). Additionally, a constant potential-independent thickness increase of 0.8% over the duration of the measurement was observed.

### Force curves & elastic modulus

**Fig. 5:**
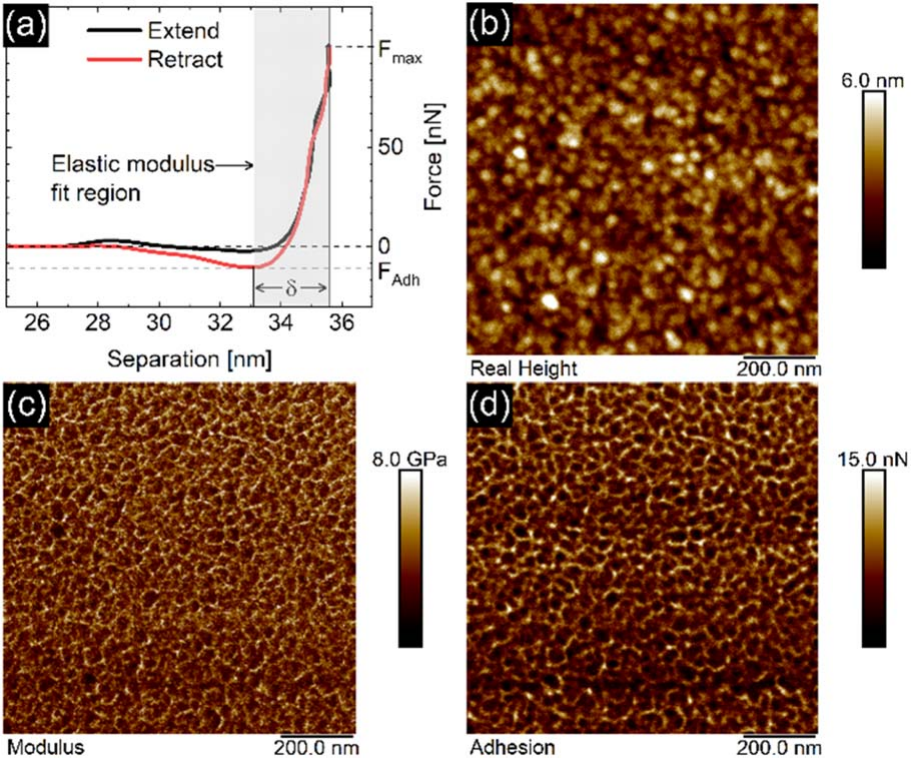
(a) Force–distance curve recorded on an aged PPy in air with a setpoint of 100 nN peak force, consisting of an extend and retract part. The maximum force setpoint *F*_max_, the adhesion *F*_adh_, and indentation *δ* are shown. The grey area denotes the fit region used for the indenter model to calculate the elastic modulus. (b) Height, (c) elastic modulus, and (d) adhesion maps calculated from the recorded force curves at every pixel in a 256 × 256 pixel QNM AFM image.

The EC-QNM AFM mode employed during the *in situ* experiments is specifically designed for the nanomechanical determination of elastic properties and the topography of sample surfaces.^20,22^ By performing a fast approach–retract cycle of the cantilever at every pixel of the image, the bending of the cantilever caused by indentation probing of the sample surface can be converted into force–distance curves as shown in Fig. 5a. These fast-recorded force curves are then analyzed on-the-fly, achieving high-resolution mapping of sample topography and elastic properties (Fig. 5b–d) of the sample.

Before analysis, a suitable indenter model is chosen. Assuming a spherical indenter for small indentations and tip–sample adhesion, the Derjaguin, Muller, and Toporov (DMT) model was used for the calculation of elastic modulus.^18,19^ The formula is given below
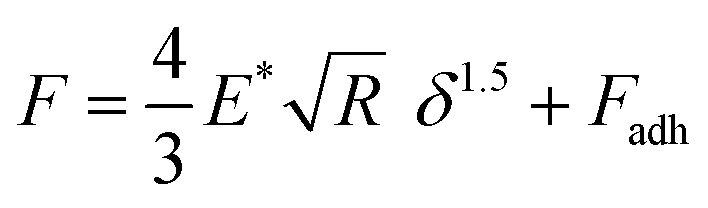
with force *F*, the reduced elastic modulus E*, the tip radius *R*, indentation *δ*, and the tip–sample adhesion *F*_adh_. The model was applied to the retract part of the force–distance curves. The reduced elastic modulus E* is related to the elastic modulus and Poisson's ratio of both indenter tip and PPy sample as shown below:
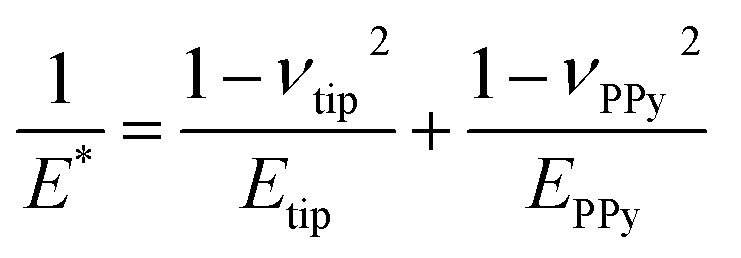


Our results for the *in situ* experiments are normalized with respect to the first data point, since no calibration of the cantilever could be performed in liquid. As such, we report the relative changes in E*.

**Fig. 6:**
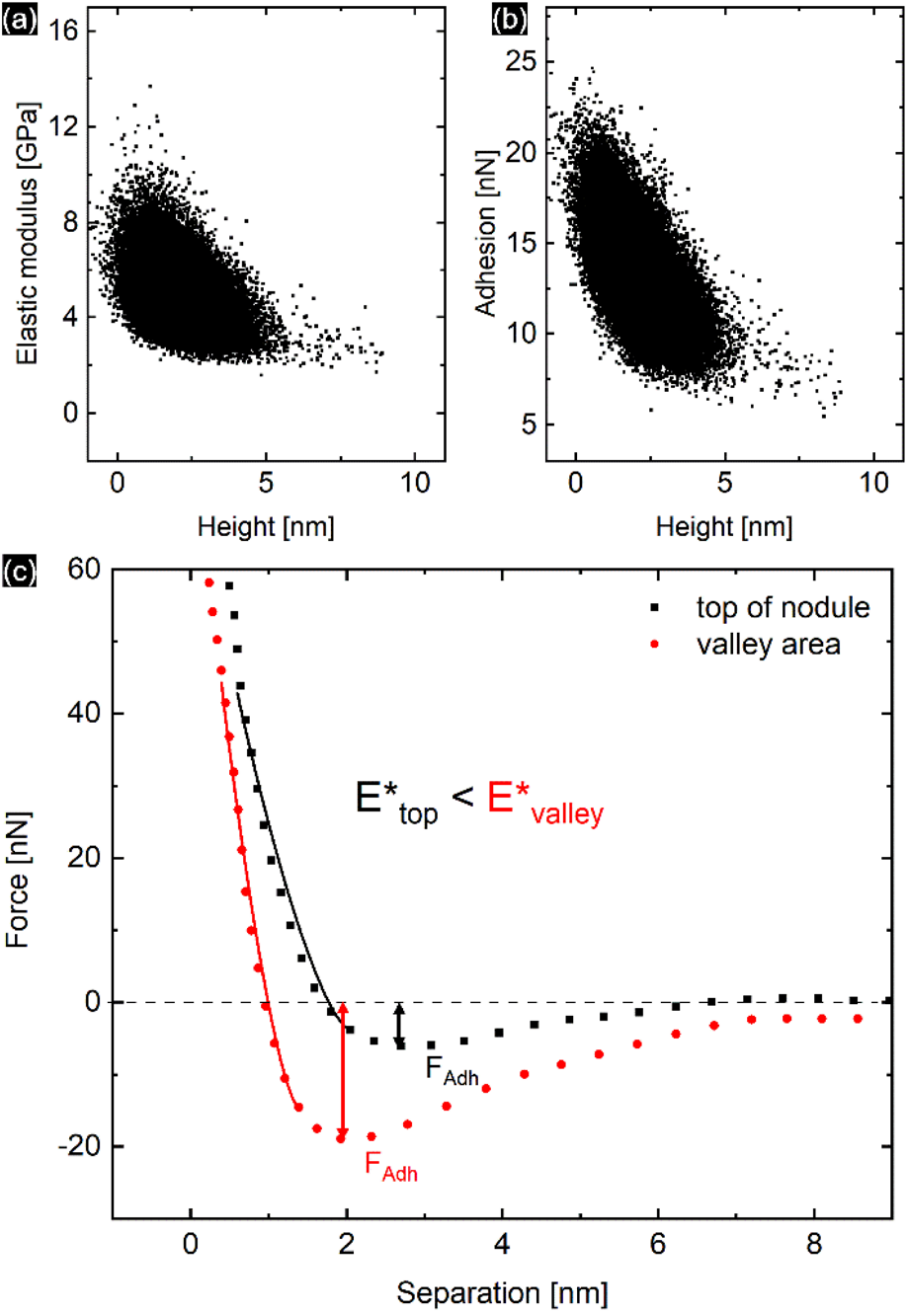
Elastic modulus (a) and adhesion force (b) taken at every pixel of the elastic property maps shown in Fig. 5 and plotted against the height of the corresponding pixels, showing a clear correlation between the two elastic properties of the aged PPy film. (c) Comparison of the retract parts of two different force curves, one from the top of the nodular areas, and the second one from the surrounding lower periphery. The solid lines represent the DMT indenter model fits for the elastic moduli, while the minimum force is defined as tip–sample adhesion force.

The PPy film shown in Fig. 5 reveals a heterogeneous distribution of its elastic modulus, with the top nodules of the cauliflower-like topography possessing a lower E* and lower adhesion than the periphery. This correlation is shown in Fig. 6a and b, where the modulus and adhesion are plotted against the measured height at every pixel of Fig. 5. A clear relationship between modulus and adhesion is visible, suggesting that the protruding top of the nodular cauliflower-like structures is not only softer (lower modulus), but shows also lower adhesion to the tip than the lower valleys of the PPy microstructure. Comparison of selected force curves from both the top of the nodules and the surrounding periphery in Fig. 6c confirm the observed trends from the pixelwise consideration in Fig. 6a and b, and clearly reveals the differences in adhesion and, from the slope of the tip–sample contact area, elastic modulus.

Fig. 3b and c show the time and potential dependent evolution of the average E* and its distribution during the static method measurement of the polypyrrole area illustrated in Fig. S5.† Along with a film thickness increase with increasing potential, the average E* rises up to 80% of its original value, while the distribution of modulus widens. However, with the dynamic method presented in Fig. 4b, potential induced E* changes of only 15% were observed. Additionally, over the duration of the dynamic experiment a passive, potential-independent softening of the PPy film was observed.

## Discussion

### EC-QNM AFM – height

It is well established that when perchlorate doped PPy is oxidized at higher potentials, ions from the electrolyte move into the polymer matrix for charge compensation.^4,6,13,29,30,36,37^ Oxidation of perchlorate-doped PPy films leads to expansion due to influx of perchlorate anions and solvent as reported by the literature and observed in Fig. 3a and 4a.^3,13,27,38^ The film thickness in Fig. 3a follows the potential nearly linearly, increasing as the potential increases. This indicates that the ion diffusion during the redox cycling is anion-prevalent. As the PPy backbone is oxidized, negatively charged perchlorate anions diffuse into the polymer matrix, effectively leading to an increase in film thickness. The observed deviation of the linear relationship between potential and PPy thickness at 0.0 V in Fig. 3a could be caused by the film not having reached an equilibrium at the point the measurement was taken. Another possibility could be mixed ionic transport contributing to the film thickness changes at lower potentials, as it has been observed before.^2,6,29^ Lakner *et al.* reported a change in PPy film behavior during electrochemical cycling in perchloric acid with XRR. They concluded that in the lower potential regime charge compensation is primarily driven by proton transfer due to anion depletion of the polymer matrix. This likely stems from electrostatic reasons, as well as low perchlorate concentration in the polymer matrix at reducing potentials, leading to an influx of protons, or as in our case, sodium cations for charge compensation.^6^

The extent of swelling depends mostly on the internal PPy structure, which is largely influenced by the synthesis. Additional factors include the pH value and the concentration and type of the electrolyte salt.^4,6,13,29,30,36,37^ PPy films synthesized under similar conditions as ours were reported to have a much lower swelling ratio when electrochemically cycled in perchloric acid.^6^ It was noted however, that in a neutral aqueous sodium perchlorate solution the swelling capacity of PPy can be significantly higher, since no protons can participate in the charge compensation.^6,30,39,40^

The smaller swelling associated with the dynamic method (2.5–3.0% V^−1^) as compared to the static method (17% V^−1^) can be explained by the much shorter timescale of the measurement. Scanning techniques like AFM are limited by the scanning speed and image resolution during the data acquisition. While intrinsic contributions to the volume expansion, such as changes in polymer backbone bond lengths due to redox switching, happen on a timescale of a few hundred milliseconds for thin films (<100 nm),^9^ a substantial part of volume expansion can be attributed to osmotic expansion with diffusion of solvent and ions into and out of the polymer matrix.^35^ The diffusion of solvent and ions and the accompanying rearrangement within the polymer take place over a longer timescale, as can be observed by the continuous increase of the film thickness slope in Fig. 4a. This potential-independent swelling has also been observed by others.^6,27,40^ It is related to long-term relaxation processes within the polymer network as well as osmotic effects, causing the film to become thicker and less dense per cycle.^6^ Compared to the actuation caused directly by the potential, this passive increase is larger than expected and could explain the discrepancies between the results of dynamic and static method, as the longer timescales in the static case lead to PPy being closer to equilibrium during the measurement. These results imply that solvent swelling of the film together with film relaxation might be more pronounced in long-term measurements. It has also been reported that the swelling and actuation of PPy films depend on the potential sweep rate frequency.^26^ For higher frequencies, the actuation decreases, which can be accounted to time-limited diffusion of the ions in the electrolyte during redox cycling, although this effect could be less pronounced in thinner PPy films as reported by Higgins *et al.*^13^

From the box-like shape of the cyclic voltammogram in Fig. S4† we conclude that the used potential range was within the capacitive potential window with no faradaic processes taking place at the electrodes. This ensured that no overoxidation took place, which would have otherwise led to irreversible degradation of the PPy film.^29^

### EC-QNM AFM – E*

QNM AFM is an operating mode for nanoindentation which oscillates the cantilever below its resonant frequency while performing fast force–distance curves on the sample.^20^ It minimizes tip wear and tip induced sample damage through force feedback control, making it an ideal tool for investigations of sensitive thin polymer films *in situ*.^41,42^

The force curve shown in Fig. 5a consists of an extend and retract cycle. When the cantilever moves in close proximity to the sample surface, attractive forces between tip and sample lead to a bending of the cantilever towards the sample until the surface is contacted (‘contact point’). As the tip indents the sample, the cantilever is increasingly stronger deflected until a maximum force setpoint is reached and the retract cycle begins. Due to tip-sample adhesion, the extend–retract cycle shows a hysteresis, with the point of minimum force being the ‘lift-off point’ where the cantilever tip ‘snaps’ off of the sample surface. Many elastic properties of the sample, such as elastic modulus and adhesion can be determined from the measured force curves. For calculation of the elastic modulus with the chosen indenter model the retract part of the force curve is used. A low indentation depth of 2.4 nm as shown in Fig. S8† is necessary to eliminate substrate effects typical for thin substrate-supported polymer films. Due to the difficult nature of cantilever calibration under *in situ* conditions necessary for the determination of absolute values of the polymer films' elastic properties, we resort to presenting relative changes in modulus for the *in situ* results in Fig. 3 and 4, and absolute values in Fig. 5 and 6 for the measurement in air with calibrated cantilevers.

As seen in Fig. 5, the elastic modulus of the PPy surface in perchlorate electrolyte is largely heterogeneous, with the top of the nodules appearing softer than their periphery. This distribution is likely caused by local structural variations of the film (*e.g.* porosity and density). We suspect that the lower elastic modulus on top of the nodules is related to the film growth mechanism during electropolymerization, leading to a larger porosity in the nodules themselves. Film porosity as well as nodular size can be fine-tuned by choice of synthesis conditions (*e.g.* solvent, ion, pH, *etc.*) and film thickness.^11^

QNM AFM holds a significant advantage over macroscopic tensile tests typically used in earlier *in situ* investigations of PPy.^13,26^ Not only does it offer additional information (*e.g.* adhesion, dissipation) about the films' elastic properties, but also achieves microscale resolution which is essential for evaluating the use of PPy systems in future applications.

Oxidation and reduction of the PPy film causes the diffusion of perchlorate anions and solvent into and out of the polymer matrix. Otero *et al.* found a linear relationship between elastic modulus and potential, which were explained by the reduction of the degrees of freedom of the polymer chains due to swelling, *i.e.*, reflecting a reduced conformational entropy.^27^ This is reflected by the increase in E* caused by oxidation of the PPy film at higher potentials seen in Fig. 3b and 4b. Similar to the film thickness, the average E* shows a substantially larger potential induced increase of nearly 80% in the static method compared to only 15% increase for the dynamic method and is accompanied by widening of the distribution of E* as shown in Fig. 3c. The larger modulus increase is attributed to the different timescales in the experiments as discussed before, while the widening of the E* distribution seems to indicate uneven or inhomogeneous swelling. From the literature, relative E* changes of up to 200% have been observed and explained by solvent swelling, ionic crosslinking, or ion diffusion.^11,36,43,44^ Also, the passive decrease of E* during the dynamic experiment in Fig. 4b indicates an additional long-term relaxation and solvent influx process, leading to an increase in film thickness coupled with softening of the film, which causes the deviation from the linear trend at higher potentials shown in Fig. 3b. The potential induced height and modulus changes in Fig. 4a and b seem unaffected by this, which hints that this passive change is caused by osmotic effects.^6,35^

### Influence of topography on elastic properties

The heterogeneous distribution of elastic modulus and adhesion of the PPy surface shown in Fig. 3c and 5 is apparently linked to the topography of the cauliflower-like surface structure. While the top of the nodular areas appears to have lower elastic modulus and low adhesion, the surrounding lower periphery possesses a comparably higher elastic modulus and adhesion. This is evident by comparing the dependence of determined elastic modulus and adhesion with the recorded height of the film as shown in Fig. 6a and b. It is likely that this effect is a combination of surface topography and internal morphology of the thin film. It has been shown that concave surface geometries can lead to an increased adhesion.^45^ Although our produced films possess remarkably low roughness, the contact area of the tip and sample might vary depending on whether the tip contacts the top of the nodules or the lower ‘valleys’, thus influencing the indenter geometry and enhancing tip–sample adhesion. There also seems to be a linear relationship between adhesion and elastic modulus, although the exact cause is unclear. Adhesion is primarily affected by the surface potentials, van der Waals forces, and capillary effects, while elastic modulus is mainly influenced by the internal morphology. However, surface roughness can lead to a correlation between both elastic properties.^46^ Whereas earlier studies did not find a systematic correlation between surface roughness and the elastic modulus of PPy films, our study has in fact revealed the top of the nodular cauliflower-like structures to be softer than the lower surrounding periphery.^26^ We assume this difference must stem from the difference in electrosynthesis conditions (*e.g.* potential control function, solvent, dopants) leading to a completely different microstructure. As it is well known, potentiodynamically deposited PPy thin films synthesized in acetonitrile and doped with compact perchlorate anions are in comparison much denser and smoother than their counterparts synthesized under different conditions.^6^ Additionally, we speculate that during electrochemical potential cycling the nodular areas facilitate solvent and dopant ion diffusion more than the lower lying surroundings as they are easier accessible for diffusion processes taking place. Repeated cycling, accompanied with the iterative insertion/extraction of solvent and ion molecules might lead to a selective “break-up” and corresponding softening of the top of the nodular areas due to changes in the PPy microstructure.

## Conclusions

In this study, a perchlorate doped thin film of PPy was potentiodynamically synthesized and investigated under *in situ* conditions with EC QNM AFM. The film thickness as well as the Young modulus of the PPy film were monitored as a function of the electric potential over different timescales *via* fast recorded force–distance curves. Film thickness and elastic modulus follow the potential, both increasing/decreasing as the film is oxidized/reduced due to solvent and ion diffusion. Even though our potentiodynamic synthesis protocol typically produces thin PPy films with a nominal low porosity compared to other synthesis protocols, we observed a swelling ratio of up to 17% V^−1^ and changes in elastic modulus of up to 80%, which were accompanied by a significant potential-independent film swelling coupled with a decrease in modulus over the duration of the experiment. This passive creep can be attributed to osmotic expansion. The high resolution 2D maps of PPy topography and elastic properties recorded with QNM AFM enabled a correlative analysis connecting the PPy film topography with local elastic modulus values and adhesion. The comparison revealed that the higher areas of the PPy nodules possess a lower elastic modulus and tip adhesion force then the surrounding lower periphery, which we assign to the interplay of the internal morphology (*e.g.* porosity) and the convex–concave cauliflower-like surface structure of the PPy. Therefore, EC QNM AFM shows a clear advantage over classic macroscopic tensile tests used for polymers by providing a microscopic view on the link between surface structure and elastic properties. Our findings highlight the versatility of AFM, enabling measurements of sample topography and its microscopic elastic properties under *in situ* conditions, thus opening a pathway to the development of novel soft electronic materials. Connecting topography with elastic properties and understanding their interdependence is a major component for future material development.

## Conflicts of interest

There are no conflicts to declare.

## Supplementary Material

NA-006-D3NA00611E-s001
