# Supplementary material for: Mapping the nanoscale elastic property modulations of polypyrrole thin films in liquid electrolyte with EC-AFM
Source: Nanoscale Adv. 2023 Nov 22;6(1):102–10. doi: 10.1039/d3na00611e (PMC10729878; doi:10.1039/d3na00611e)
Supplement: NA-006-D3NA00611E-s001 [file NA-006-D3NA00611E-s001.pdf]

Electronic Supplementary Material (ESI) for Nanoscale Advances.
This journal is © The Royal Society of Chemistry 2023

Mapping the nanoscale elastic property modulations of polypyrrole thin films in liquid electrolyte with EC-AFM

*Alexander Meinhardt[1,2], Pirmin Lakner[1,2], Patrick Huber[1,3,4], Thomas F. Keller[1,2]**

[1]Centre for X-ray and Nano Science (CXNS), Deutsches Elektronen-Synchrotron DESY, Hamburg, Germany

[2]Department of Physics, University of Hamburg, Hamburg, Germany

[3]Hamburg University of Technology, Institute for Materials and X-Ray Physics, Hamburg, Germany

[4]Center for Hybrid Nanostructures CHyN, Hamburg University, Hamburg, Germany

Keywords: polypyrrole, conductive polymer, electropolymerization, in situ EC-AFM, Young modulus, elastic properties, electrochemistry

Supporting Information

Details of the electrochemical cell used for electropolymerization, the in situ AFM setup, cyclic voltammogram and complementary AFM data.

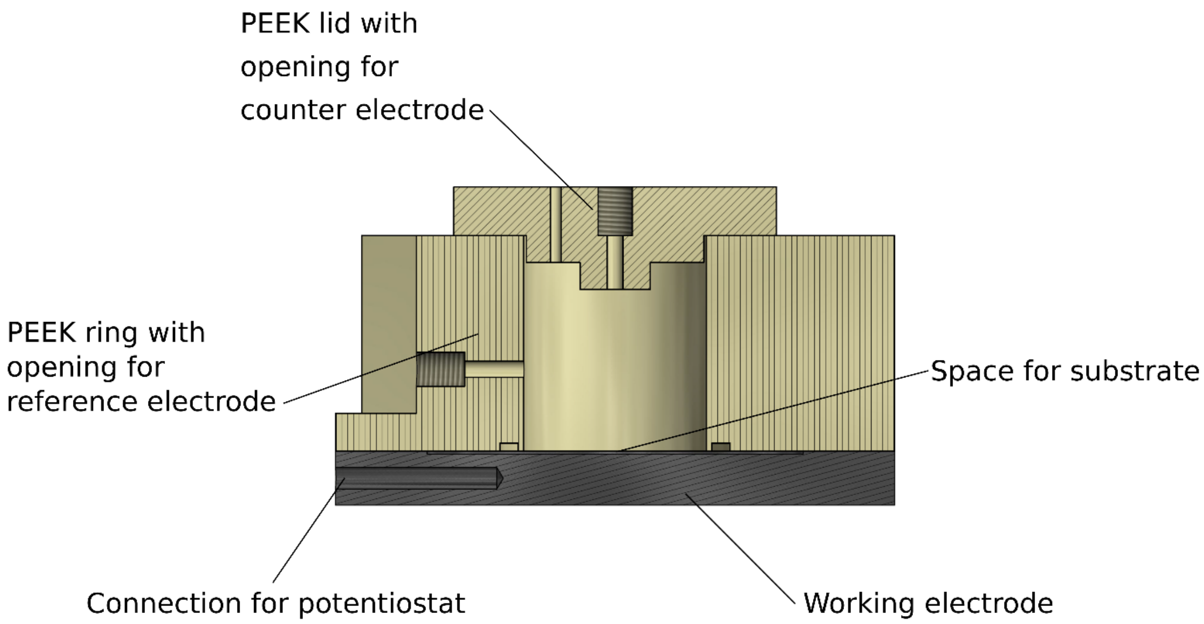

Figure S1. EC cell used for electropolymerization of PPy thin film.

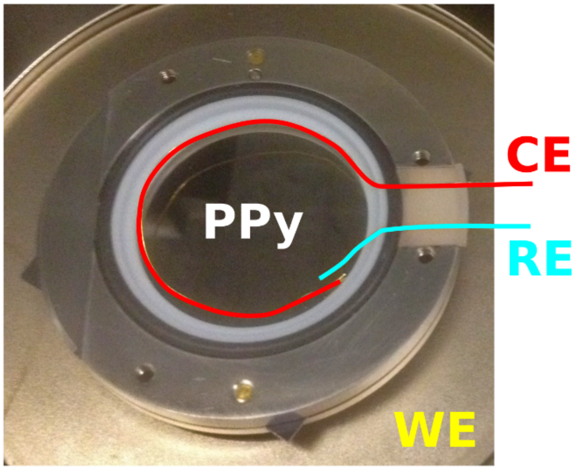

Figure S2. Fluid AFM cell with electrode arrangement used for the *in situ* electrochemistry experiments.

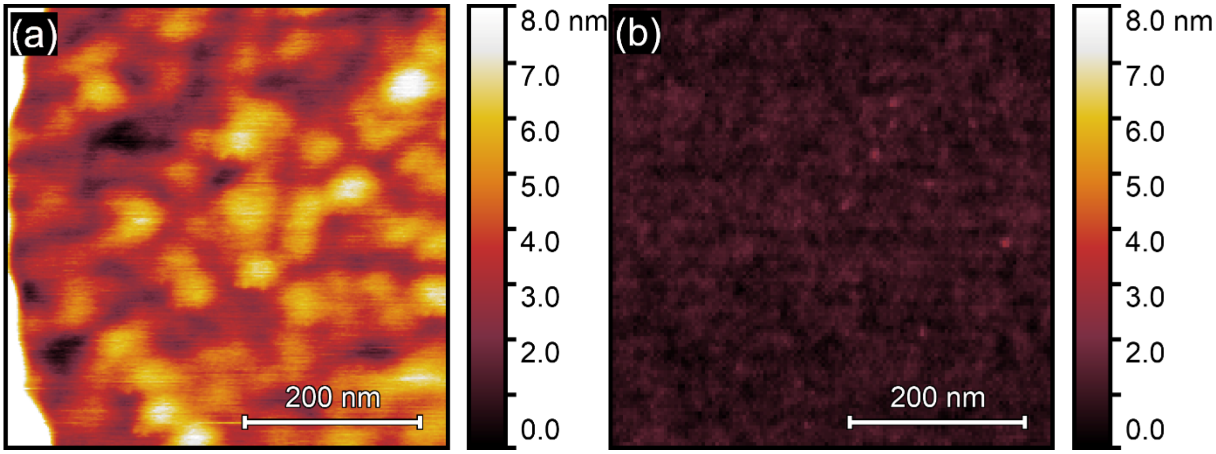

Figure S3. AFM tapping mode height image in air showing the surface roughness of substrate area that was previously covered by PPy (left). Some residual PPy may cause the roughness. Clean Si substrate without any PPy showing clearly lower surface roughness (right).

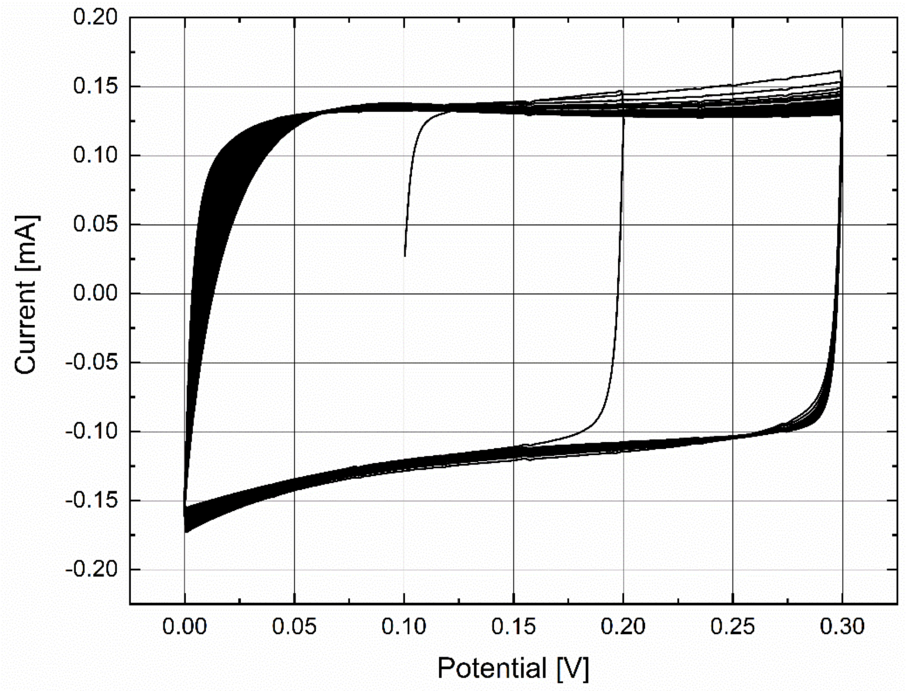

Figure S4. Cyclic voltammogram of PPy film ranging from 0.0 V to 0.3 V with a potential cycling rate of 10 mV/s for 50 cycles during the *in situ* EC AFM experiments. The box-like shape indicates that no faradaic processes or degradation take place within the chosen potential window.

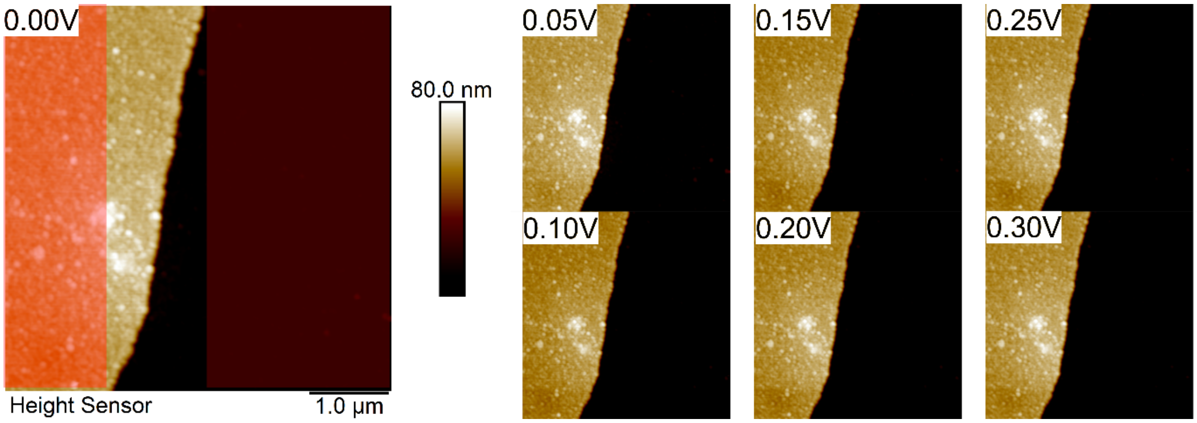

Figure S5. AFM images taken at every potential step for the static method. The average film thickness is determined from the marked areas.

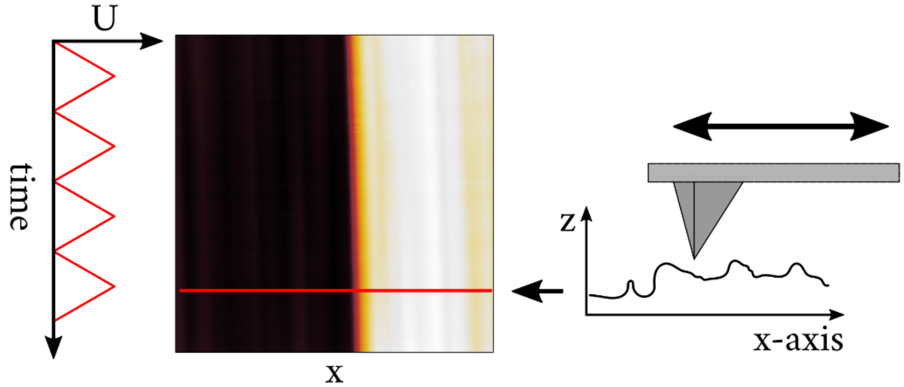

Figure S6. Schematic image illustrating how the dynamic method works. The AFM tip scans repeatedly over the same line while the electrical potential is varied.

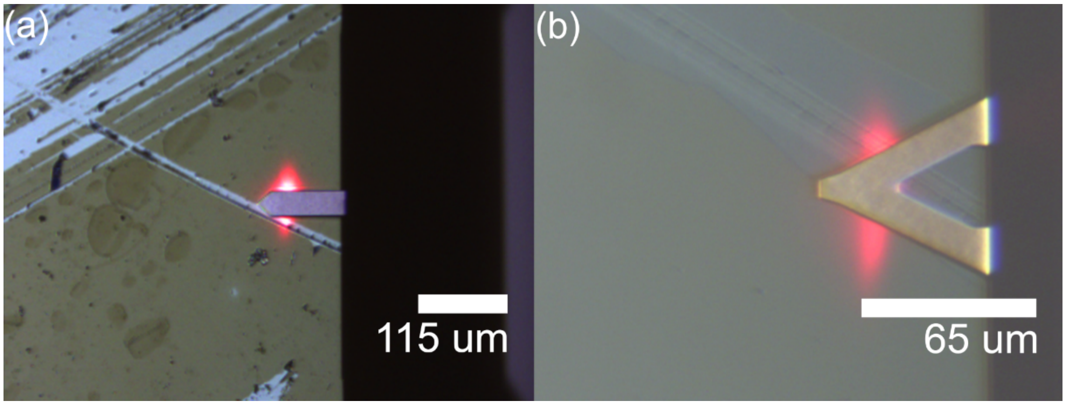

Figure S7. Optical microscopy images recorded by the build-in optical microscope of the AFM. In (a), a rectangular silicon tapping mode cantilever (RTESPA-300, Bruker) scans across a scratch of the polypyrrole film. In (b), a similar scan is performed in fluid using a triangular silicon nitride cantilever (SCANASYST-FLUID+).

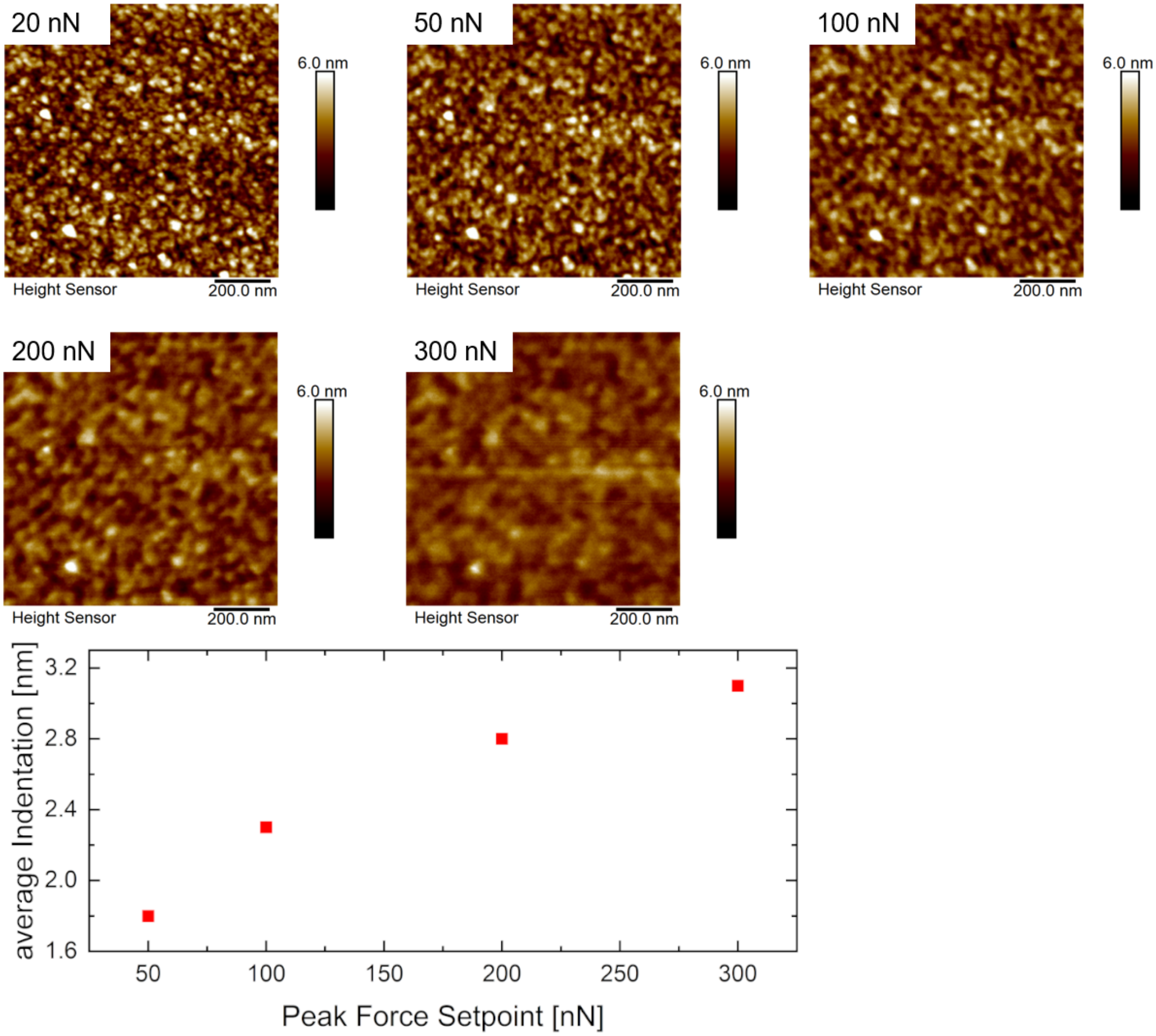

Figure S8. QNM-AFM images of the polypyrrole film topography measured at different maximum force setpoints. The higher force leads to larger indentation depth, but also clearly damages the film.
